# Novel Pre- and Postoperative Care Using Telemedicine

**DOI:** 10.3389/fsurg.2020.596970

**Published:** 2020-12-01

**Authors:** Dana Ferrari-Light, Travis C. Geraci, Stephanie H. Chang, Robert J. Cerfolio

**Affiliations:** ^1^Department of Surgery, New York Presbyterian-Queens, Flushing, NY, United States; ^2^Department of Cardiothoracic Surgery, NYU Langone Health, New York, NY, United States

**Keywords:** telemedicine, thoracic surgery, outcomes, enhanced recovery, telehealth, outpatient activity

## Abstract

The use of telemedicine and telehealth services has grown exponentially over the past decade and has become increasingly relevant and necessary during the coronavirus 2019 (COVID-19) pandemic. There remains ample opportunity to electronically connect cardiothoracic surgeons with their patients during both preoperative and postoperative visits. In this review, we examine the various implementations of telemedicine within thoracic surgery and explore future applications in this quickly developing field.

## Introduction

Telemedicine (also referred to as telehealth) is a general term that encompasses any form of electronic communication in medicine, whether between clinicians, clinician to patient, or patient interaction with mobile health technology. These tools and services are becoming increasingly popular in the United States (US) and around the world. As of 2019, 76% of US hospitals used some form of telehealth to connect with patients and providers through digital and video technology (1). Due to the recent COVID-19 pandemic, the US Center for Medicare and Medicaid Services (CMS) greatly expanded its previously limited coverage for telemedicine visits, which has skyrocketed the development and use of telehealth platforms (2).

The surgical specialties have been rapid adopters of telemedicine compared to other medical specialties, such as radiology, pathology, neurology and psychiatry practices (3–5). The ultimate goal for surgeons and surgical patients utilizing telemedicine is to minimize in- office preoperative time, while maintaining the adequacy and safety of assessing preoperative patient fitness for surgery. An additional benefit is to reduce the need for the total number of postoperative office and emergency department visits. As we have shown in our previous experience, perioperative telemedicine use in patients undergoing major thoracic surgery is a safe and effective means of communication and care and has significant travel, cost, and time savings without compromising clinical outcomes (6).

## Thoracic Surgery

Telehealth services are becoming increasingly popular within the realm of thoracic surgery in all aspects of patient care: preoperative consultation, surgical planning and fitness testing, and postoperative follow-up. Preoperative imaging, work-up, and consultation for thoracic surgical patients can be expedited and streamlined via telemedicine. A 2018 study from the US Veterans Affairs (VA) system developed a virtual consultation system for veterans with pulmonary nodules who were finding it difficult to access specialty thoracic care (7). Compared to veterans who underwent traditional in-office visits, veterans who completed a virtual consultation had a greatly reduced mean time to consultation (2.8 vs. 20.5 days), a 47.4% decrease in VA hospital costs, and the complete elimination of travel costs (of note, veterans lived an average of 86 miles away from the VA medical center). A similar study performed between 2003 and 2015 in British Columbia, Canada connected thoracic surgeons to patients living in remote areas via “virtual thoracic surgical clinics.” This study promoted the “hub and spoke” model of care in which remote patients can access centralized services and saved them nearly 11.5 million km (over 7 million miles) of travel distance to care, with an average savings of 766 km (roughly 475 miles) per person (8).

The advantages of telemedicine expand into the postoperative care of the thoracic patient as well. In 2011, Cleeland et al. used an automated telephone questionnaire to monitor postoperative symptoms and pain in patients who underwent pulmonary resection via open thoracotomy for lung cancer or metastases (9). Frequent symptom telemonitoring for patients was combined with email alerts to clinicians if moderate or severe postoperative symptoms (pain, sleep disturbance, dyspnea, distress, or constipation) were reported in the first 4 weeks after surgery. When compared to the control group who generated no email alerts, patients in the study group reported a larger reduction and more rapid decline in moderate or severe symptom events. Both patients and clinicians in this study expressed satisfaction with the automated telemedicine survey system and postoperative symptom control.

Postoperative chest tube management has also been streamlined by telemedicine and tele- monitoring systems. Digital chest tube drainage systems can reduce the risk of postoperative air leak, reduce treatment time and shorten hospital stays by the ability to monitor chest tube parameters wirelessly distant from the patient (10, 11). An additional benefit of this system is the increased patient convenience which provides satisfaction and confidence in the system - which has been replicated at multiple surgical centers around the world (12). Digital drainage systems for patients who are discharged with a chest tube also allows for patients to have their tubes removed precisely when their air leak stops since they can read when the digital chamber has reached a zero value (indicating cessation of air-leak). This objective measurement allows patients to report daily data via telephone and to present to clinic to have their chest tube removed on the day of resolution, rather than waiting to schedule an appointment for the clinician to subjectively evaluate the tube and air leak.

Our own research has examined the safety and feasibility of using telemedicine both in the preoperative and postoperative phase of patient care. Our prospective study of 56 patients undergoing major thoracic surgery who had both pre- and postoperative telemedicine visits showed maintenance of excellent outcomes with no major morbidity or mortalities (6). Unscheduled telemedicine visits prevented six unnecessary emergency room visits. Telemedicine was implemented while maintaining high patient satisfaction scores. Anecdotally, we found that preoperative assessment via telemedicine allowed an opportunity to evaluate the patient and patient-environment. Specifically, we were able to assess functional limitations such as number of stairs in the house or the height of the bathtub, as well as observe the level of social and/or familial support at home. Any potential barriers to safe recovery at home are frequently identified and addressed prior to surgery.

One of the most exciting and rewarding aspects of telemedicine in thoracic surgery is the ability to support and promote a healthy postoperative recovery phase for patients undergoing lung resection. Telehealth services that include web-based and smartphone apps that are accessible on-demand to patients have been successful in promoting postoperative recovery through patient motivation and positive reinforcement (13, 14). A 2017 study in the Netherlands examined the effects of a preoperative and postoperative ambulant home monitoring system and web-based home exercise plan for lung cancer patients who underwent lung resection (14). Patients in the study perceived these telehealth programs to be beneficial and contributed to their recovery after surgery. Interestingly, the involvement of health care practitioners with this program varied greatly – with physiotherapists reporting high frequency of use whereas physicians hardly used the data collected from the program in their postoperative consultations.

This highlights the importance of “perceived usefulness” and the importance of integrating new or existing telehealth systems in a way that facilitates or improves existing provider workflow.

## The COVID-19 Pandemic

The global COVID-19 pandemic has rapidly overwhelmed the medical system of many countries, including the United States. Thoracic oncology patients are at an advanced risk of morbidity and mortality from this novel coronavirus, especially if they are exhibiting respiratory symptoms or undergoing radiotherapy or chemotherapy when they become infected (15–17). Per current US Center for Disease Control (CDC) guidelines, these high-risk patients should be evaluated by thoracic surgeons and oncologists via telemedicine when possible (18–20).

A recent publication from Jefferson Health in 2020 describes their division of thoracic surgery's almost complete transition to telemedicine outpatient visits in response to the COVID- 19 pandemic, including both preoperative and postoperative evaluations (21). Although patient outcomes and satisfaction were not measured, similar reorganization of hospitals and outpatient clinics elsewhere in the US have made it clear that telemedicine will need to be incorporated into thoracic surgical practice for the foreseeable future to ensure the safety of patients, clinicians, and all supportive staff (22).

## Discussion

The advantages of incorporating telemedicine into thoracic surgical practice are myriad. For patients, telehealth visits cut down on transportation costs, do not require time off work, prevent childcare issues, decrease exposure to disease in the community, allow for patient access to specialty and subspecialty providers over great geographic distances, and improve patient engagement with clinicians. For clinicians, the benefits include improved workflow and ability to see distant patients, increased practice revenue and decreased overhead, and quicker access to specialty providers for consults and referrals. In a global pandemic, telehealth services provide opportunity to flatten the transmission curve to allow for well and mildly ill patients to remain at home.

However, this exciting emerging component of healthcare does not come without caveats. There is a risk of perpetuating socioeconomic disparities if patients do not have access to, or cannot afford, the technology required to engage in telemedicine visits. While there is currently expanded coverage for telehealth services in the US due to the COVID-19 pandemic, it is unclear if these rules will remain permanently and may limit both patient coverage and provider reimbursement for care. Finally, there are certain testing, physical exams, and rapport- building interactions in an office visit which cannot be replicated with a video or telephone call, and thus the patient-physician relationship may suffer.

There is no doubt that telemedicine is a permanent addition to the medical and surgical landscape. It is crucial that today's thoracic surgeon embraces further innovations and succeeds in adapting new technology into clinical workflow. Research opportunities are limitless in this area however high-quality, evidence-based research needs to expand and confirm the benefits of telemedicine that are extolled in smaller studies. Finally, telemedicine represents the opportunity to expand care to those that need it most – the patients who have limited access to physicians and comprehensive healthcare, especially the exposure to surgical subspecialties. When implementing or evaluating a telehealth system, patient access, outcomes and economic factors must be top priority. Clinical, organizational, and research teams should work in tandem to ensure that telemedicine is a safe, affordable, and beneficial modality for all patient to access care.

## Author Contributions

DF-L, TG, SC, and RC contributed equally to the formation, writing, and editing of this manuscript. All authors contributed to the article and approved the submitted version.

## Conflict of Interest

DF-L discloses compensation for database entry for an unrelated project from Intuitive Surgical. RC discloses relationships with AstraZeneca, Bard Davol, Bovie Medical Corporation, C-SATS, ConMed, Covidien/Medtronic, Ethicon, Fruit Street Health, Google/Verb Surgical, Intuitive Surgical, KCI/Acelity, Myriad Genetics, Neomend, Pinnacle Biologics, ROLO-7, Tego, and TransEnterix. The remaining authors declare that the research was conducted in the absence of any commercial or financial relationships that could be construed as a potential conflict of interest.
